# Ascorbic acid does not enhance hypoxia‐induced vasodilation in healthy older men

**DOI:** 10.14814/phy2.12091

**Published:** 2014-07-22

**Authors:** Jonathan P. Pollock, Hardikkumar M. Patel, Brittney J. Randolph, Matthew J. Heffernan, Urs A. Leuenberger, Matthew D. Muller

**Affiliations:** 1 Penn State Hershey Heart and Vascular Institute, Pennsylvania State University College of Medicine, Hershey, Pennsylvania, USA

**Keywords:** Antioxidant, chemoreflex, forearm blood flow, sympathetic nervous system, vascular resistance

## Abstract

In response to hypoxia, a net vasodilation occurs in the limb vasculature in young healthy humans and this is referred to as “hypoxia‐induced vasodilation”. We performed two separate experiments to determine (1) if hypoxia‐induced forearm vasodilation is impaired in older men (*n* = 8) compared to young men (*n* = 7) and (2) if acute systemic infusion of ascorbic acid would enhance hypoxia‐induced vasodilation in older men (*n* = 8). Heart rate, mean arterial pressure, oxygen saturation, minute ventilation, forearm vascular conductance (FVC, Doppler ultrasound), and cutaneous vascular conductance (CVC, laser Doppler flowmetry) were recorded continuously while subjects breathed 10% oxygen for 5 min. Changes from baseline were compared between groups and between treatments. The older adults had a significantly attenuated increase in FBF (13 ± 4 vs. 30 ± 7%) and FVC (16 ± 4 vs. 30 ± 7%) in response to 5 min of hypoxia. However, skin blood flow responses were comparable between groups (young: 35 ± 9, older: 30 ± 6%). In Experiment 2, FVC responses to 5 min of breathing 10% oxygen were not significantly different following saline (3 ± 10%) and ascorbic acid (8 ± 10%) in the older men. Ascorbic acid also had no physiological effects in the young men. These findings advance our basic understanding of how aging influences vascular responses to hypoxia and suggest that, in healthy humans, hypoxia‐induced vasodilation is not restrained by reactive oxygen species.

## Abstract (precis)

e12091

In Experiment 1, heart rate (HR), mean arterial pressure (MAP), forearm vascular conductance (FVC), and forearm cutaneous vascular conductance (CVC) were measured in response to 5 min of continuous hypoxia (10% oxygen) in young men (black diamonds) and older men (white squares). In Experiment 2, older men breathed continuous hypoxia after receiving normal saline solution (NSS, white squares) and ascorbic acid (Vit C, black squares). These findings advance our basic understanding of how aging influences vascular responses to hypoxia and suggest that, in healthy humans, hypoxia‐induced vasodilation is not restrained by reactive oxygen species.

## Introduction

Hypoxemia is a hallmark of several disease states including obstructive sleep apnea (OSA), chronic obstructive pulmonary disease (COPD), and acute mountain sickness and is also experienced during recreational activities (e.g., diving, hiking). In these conditions, maintaining (or restoring) oxygen delivery to skeletal muscle, cardiac muscle, and the brain is fundamentally important or impaired performance and eventual tissue necrosis may ensue. Human experiments have shown that hypoxemia raises ventilation, heart rate, and muscle sympathetic nerve activity (Saito et al. 1988; Rowell et al. 1989; Somers et al. 1989). An increase in muscle sympathetic nerve activity helps to redistribute blood flow to vital organs. Despite this acute rise in vasoconstrictor nerve traffic to the resting limb, a net vasodilation occurs in both skeletal muscle and the skin in young healthy subjects (Heistad and Wheeler 1970; Kravec et al. 1972; Leuenberger et al. 1991; Moradkhan et al. 2007; Simmons et al. 2007). The net vascular effects are referred to as “hypoxia‐induced vasodilation” and this process is important to enhance oxygen delivery to tissues. Previous studies have demonstrated a 20–35% increase in forearm blood flow (FBF) (Leuenberger et al. 1999; Weisbrod et al. 2001), a 25–35% increase in femoral blood flow (Leuenberger et al. 2001; DeLorey et al. 2004) and a 15–30% increase in forearm cutaneous vascular conductance (CVC) (Simmons et al. 2007) in healthy people age 18–33. However, the influence of healthy aging on hypoxia‐induced vasodilation (both muscle blood flow and skin blood flow) is largely unknown.

Older adults (age 50–75) experience a blunted tachycardia in response to hypoxia compared to young subjects (Kronenberg and Drage 1973; Lhuissier et al. 2012) whereas differences in ventilation (Lhuissier et al. 2012; Muller et al. 2013b), blood pressure (BP), and muscle sympathetic nerve activity (MSNA) are not consistently observed. Indeed, Davy et al. (1997) demonstrated that the increases in MSNA and BP were comparable between young (24 ± 1 years) and older subjects (age 64 ± 1 years). When considering the vascular responses to hypoxia, two studies have demonstrated age‐related impairments in forearm blood flow (Kravec et al. 1972; Kirby et al. 2012) but two other studies did not observe differences between groups (Casey et al. 2011; Limberg et al. 2012). Importantly, none of these studies specifically evaluated skin blood flow responses to hypoxia. Thus, further work in this area is warranted.

The magnitude of hypoxia‐induced vasodilation is partly dependent on endothelial function as well as other local and systemic factors including activation of beta‐ adrenergic receptors (Richardson et al. 1967; Richards et al. 2013), adenosine (Carlsson et al. 1987; Leuenberger et al. 1999), prostaglandins (Carlsson et al. 1987; Engelke et al. 1996), release of ATP from red blood cells (Kirby et al. 2012), and local myogenic factors (Carlsson et al. 1987; Schubert and Mulvany 1999; Koller and Bagi 2002). Because endothelial function clearly declines with age (Celermajer et al. 1994; Eskurza et al. 2004; Newcomer et al. 2005; Holowatz et al. 2007), it is possible that hypoxia‐induced vasodilation would also be attenuated in older adults. One established concept is that aging is associated with increased levels of oxidative stress, which inactivates endothelial nitric oxide (Gryglewski et al. 1986; Katusic and Vanhoutte 1989) thereby causing impairments in endothelium‐dependent vasodilation (Taddei et al. 1998). Previous human studies have demonstrated that impairments in vascular function due to the aging process can be acutely modified by infusion of high dose ascorbic acid (i.e., a powerful antioxidant capable of scavenging oxygen‐derived free radicals) (Frei 1991; Eskurza et al. 2004; Monahan et al. 2004; Kirby et al. 2009). In other words, acute infusion of an antioxidant can enhance vascular function in groups of subjects with enhanced oxidative stress (e.g., older adults). However, it remains to be tested whether oxidative stress influences the acute physiological responses to hypoxia and whether there are differences between skin and muscle blood flow.

With this background in mind, the purpose of the current study was to determine if hypoxia‐induced forearm vasodilation is impaired in older men compared to young men (Experiment 1) and to determine if acute systemic infusion of ascorbic acid would enhance hypoxia‐induced vasodilation in older men (Experiment 2). We hypothesized that older men would display a decreased forearm vasodilator response (Doppler ultrasound and laser Doppler flowmetry) to 5 min of hypoxia compared to young healthy men (Experiment 1) and that ascorbic acid would enhance hypoxia‐induced vasodilation in the older men (Experiment 2).

## Methods

### Experimental design and subjects

Experiment 1 used a repeated measures design, between‐subjects design where physiological responses to hypoxia (base, 1, 2, 3, 4, 5 min) were compared between young and older subjects. Experiment 2 used a two treatment (saline, ascorbic acid), repeated measures design and enrolled both young and older men.

All study protocols were approved in advance by the Institutional Review Board of the Penn State Milton S. Hershey Medical Center and conformed to the Declaration of Helsinki. A total of seven young (26 ± 1 years, range 23–29 years) and eight older men (63 ± 1 years, range 55–81 years) participated in Experiment 1 and provided written informed consent. In a similar way, five young men and eight older men participated in Experiment 2 (demographics in Table 1). Two young men and three older men participated in both Experiments 1 and 2; control trials were repeated in these subjects. Women were excluded due to established differences in vascular responses to chemoreflex activation (Casey et al. 2013; Patel et al. 2014). All participants had supine resting BP below 130/80 mmHg and all were nonasthmatic, nonobese, nonsmokers, not taking any prescription or vasoactive medication, and were in good health as determined by history and physical examination. All participants reported being physically active but none were competitive athletes. Participants refrained from caffeine, alcohol, and exercise for 24 h before the study. All trials were conducted in the morning, following an overnight fast. Some of the subjects reported to be taking multivitamins but none were consumed on the morning of the study.

**Table 1.** Anthropometric and baseline hemodynamic characteristics.

	Young	Older
Age (years)	28 ± 1	63 ± 3*
Height (m)	1.81 ± 0.02	1.79 ± 0.01
Weight (kg)	81.8 ± 3.5	86.7 ± 3.0
BMI (kg/m2)	24.9 ± 0.7	26.9 ± 0.7
SBP (mmHg)	105 ± 4	114 ± 3
DBP (mmHg)	52 ± 2	75 ± 3*
MAP (mmHg)	69 ± 1	87 ± 2*
HR (beats/min)	56 ± 4	57 ± 4
Triglycerides (mg/dL)	67 ± 3	70 ± 9
Cholesterol (mg/dL)	132 ± 13	159 ± 8
HDL (mg/dL)	48 ± 8	44 ± 4
LDL (mg/dL)	70 ± 6	102 ± 8*
hs‐CRP (mg/dL)	0.3 ± 0.1	1.6 ± 0.4*
Fibrinogen (mg/dL)	207 ± 8	286 ± 8*
Ascorbic acid (mg/dL)	0.8 ± 0.2	0.9 ± 0.1

BMI, body mass index; SBP, systolic blood pressure; DBP, diastolic blood pressure; MAP, mean arterial pressure; HR, heart rate; HDL, high‐density lipoprotein; LDL, low‐density lipoprotein; hs‐CRP, high sensitive C‐reactive protein.

* Denotes a significant difference from young men. M ± SEM.

### Physiological measurements

All experiments were performed in the supine posture in a dimly lit thermoneutral laboratory (22–25°C). Upon arrival to the laboratory, participants dressed in a high‐density tube‐lined suit (Med‐Eng Systems, Ottawa, ON, Canada) that covered the entire body except for the feet, hands, head, and both forearms. Neutral water (34–35°C) was perfused through the suit to maintain mean skin temperature at a constant level and ensure the obtained responses were reflex mediated and not influenced by ambient temperature. After 15 min of quiet rest, baseline BP was obtained via automated oscillometry of the brachial artery (Philips SureSigns Vs3) in triplicate.

Beat‐by‐beat BP was measured by photoplethysmography (Finometer, FMS) and HR was measured via 3‐lead EKG (Cardiocap/5, GE Healthcare). Minute ventilation (MV, L/min) and end tidal CO2 (EtCO2, mmHg) were determined with a respiratory gas monitor (model RGM 5250, Ohmeda) at the expiratory port of a tight‐sealing breathing mask and SaO2 (%) was measured by ear oximetry. Cutaneous blood flow flux was measured by three laser Doppler probes (Moor Instruments, Wilmington, DE) placed on the right ventral forearm with the local temperature set at 34°C.

Beat‐by‐beat mean brachial blood flow velocity was measured in the left arm by Doppler ultrasound (Vivid 7, GE) as previously described (Wilson et al. 2007; Muller et al. 2013a). Briefly, a 9L linear transducer was placed over the left brachial artery and the insonation angle was <60°. Mean velocity was acquired in pulsed Doppler mode and velocity waveforms were synchronized to a data acquisition system (PowerLab, ADInstruments) by a Doppler audio transformer (Herr et al. 2010). Because our ultrasound does not allow for the simultaneous measurement of velocity and diameter, diameter measurements were made during hypoxia and normoxia at the following time points: 60–75, 150–165, and 270–285 s. Thus, we assumed that diameters were the same during minute 1 and 2 and also during minute 3 and 4.

#### Experiment 1: Continuous hypoxia in young men versus older men

For the hypoxia trials (10% oxygen), subjects were outfitted with an oronasal facemask for the measurement of MV and EtCO2. Baseline physiological values were obtained for ~3 min and the subjects were then switched to 10% oxygen for five additional minutes, consistent with previous studies (Leuenberger et al. 2001; Moradkhan et al. 2010; Muller et al. 2013b). The subjects were not told when the inspiratory gas concentration would be changed.

#### Experiment 2: Continuous hypoxia: saline versus ascorbic acid

**Figure 1.**
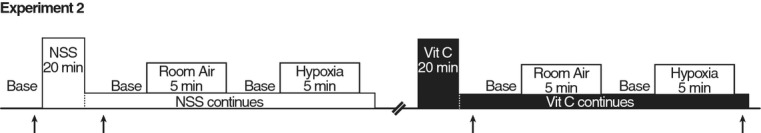
Timeline for Experiment 2. NSS = normal saline solution, Vit C = ascorbic acid, base = baseline, the arrows denote that a venous blood sample was obtained.

Based on the findings of Experiment 1, additional studies were conducted in eight older men and five young men. After dressing in the thermal suit, two intravenous catheters were placed. Following 15 min of quiet rest, baseline hemodynamic measurements were obtained. Venous blood was drawn for the measurement of cholesterol, triglycerides, high‐sensitive C‐reactive protein, and fibrinogen. These values were all determined by the Hershey Medical Center Clinical Laboratories following standard laboratory procedures. Serum ascorbic acid was measured by Quest Diagnostic Nichols Institute (San Juan Capistrano, CA). As depicted in Figure 1, saline infusion always occurred first because high dose ascorbic acid remains in the body for several hours. This experimental approach is consistent with previous reports from our laboratory (Muller et al. 2013a).

The dose of ascorbic acid was given based on the participant's body weight. A loading dose (45 mg/kg in 100 mL saline) was infused over 20 min followed by a maintenance dose (15 mg/kg in 33 mL saline) for the remainder of the study. The dose was chosen based on past human experiments in which acute intravenous ascorbic acid evoked an antioxidant physiologic effect (Eskurza et al. 2004; Monahan et al. 2004; Muller et al. 2012). Blood samples were drawn from the opposite arm of the infusion. A 10 min break occurred between infusions.

For both normoxia (21%) and hypoxia trials (10% oxygen), subjects were outfitted with an oronasal facemask for the measurement of MV and EtCO2. Baseline physiological values were obtained for ~3 min and the subjects were then switched to either 21% or 10% oxygen for five additional minutes. The subjects were not told what gas they were breathing but the researchers always administered hypoxia second since hypoxia can have long‐lasting effects (Xie et al. 2001). Both hypoxia and MVEEA trials were conducted under both saline and ascorbic acid.

### Data collection and statistical analysis

Data were collected at 200 Hz by a PowerLab (ADInstruments) and were analyzed offline, using the last 15 s of each stage. Forearm blood flow (FBF) was calculated by multiplying the cross‐sectional area (πr^2^) of the vessel by mean brachial blood flow velocity and by 60 to express FBF in units of mL/min. Forearm vascular conductance (FVC) was calculated as FBF/MAP and expressed as a percent change from baseline [(minute 5‐ baseline)/baseline × 100)], consistent with prior reports (Muller et al. 2013a). The three forearm skin blood flow sites were averaged and CVC was calculated as skin blood flow flux/MAP and then expressed as a percent change from baseline which is a common method of data presentation for CVC (Tew et al. 2012; Muller et al. 2013c,d). Changes in HR and MAP from baseline were determined in absolute physiological units.

All statistical analyses were conducted using IBM SPSS 21.0, and graphics were produced using Microsoft Excel and Adobe Illustrator CS5. Baseline anthropometric and hemodynamic parameters were determined with independent samples t‐tests (Table 1).

For Experiment 1, two group (young, older) by six time point (base, 1, 2, 3, 4, 5 min) repeated measures ANOVAs were conducted to determine the effect of age on physiological responses to hypoxia. For Experiment 2, the effects of normoxia and hypoxia were determined separately with respect to age, infusion, and time by using repeated measures ANOVA. Paired t‐tests were used when a significant group x time interaction was attained. Significance was set at *P* < 0.05 and data are presented as M ± SEM throughout.

## Results

### Experiment 1: Continuous hypoxia in young men versus older men

**Figure 2.**
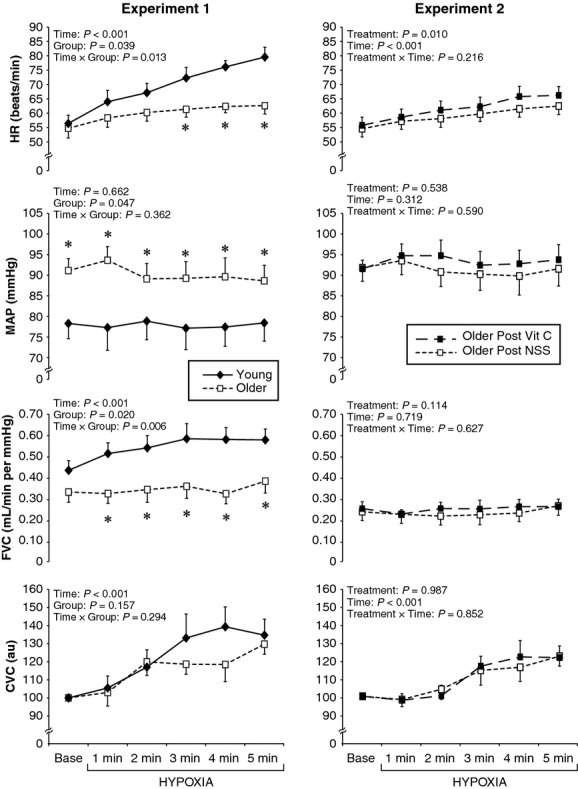
Physiologic response to continuous hypoxia. In Experiment 1, heart rate (HR), mean arterial pressure (MAP), forearm vascular conductance (FVC), and forearm cutaneous vascular conductance (CVC) were measured in response to 5 min of continuous hypoxia (10% oxygen) in young men (black diamonds) and older men (white squares). In Experiment 2, older men breathed continuous hypoxia after receiving normal saline solution (NSS, white squares) and ascorbic acid (Vit C, black squares). Data are M ± SEM, *P < 0.05 between groups at each specific time point.

In response to 5 min of breathing 10% oxygen, the young and older men had similar SaO2 (81 ± 2 vs. 81 ± 2%, *P* = 0.891). As shown in Figure 2 (left panel), the older subjects had an attenuated tachycardic response compared to the young subjects. MAP was unchanged by 5 min of hypoxia but was higher in the older men at all time points. At baseline, FVC (*P* = 0.153) and FBF (young: 34 ± 4, older: 31 ± 4 mL/min, *P* = 0.627) were similar between groups. However, the older adults had a significantly attenuated increase in FVC in response to 5 min of hypoxia (i.e., hypoxic vasodilation in skeletal muscle was blunted). Indeed, the percent change in FBF (young: 30 ± 7, older: 13 ± 4%, *P* = 0.036) and FVC (young: 30 ± 7, older: 16 ± 4%, *P* = 0.047) were attenuated in the older adults. However, skin blood flow responses were comparable between groups (young: 35 ± 9, older: 30 ± 6%, *P* = 0.636, Figure 2).

### Experiment 2: Continuous hypoxia: saline versus ascorbic acid

The groups had similar serum levels of ascorbic acid at baseline (Table 1). The young men received a total of 4908 ± 132 mg of intravenous ascorbic acid during the study and the older men received a total of 4942 ± 85 mg. Infusion of saline had no effect on serum ascorbic acid levels (young: 0.8 ± 0.2 mg/dL, older: 1.0 ± 0.1 mg/dL, *P* = 0.258). After the loading dose of ascorbic acid, serum levels of ascorbic acid drawn from the opposite arm were 15.2 ± 0.6 mg/dL in the young men and 13.6 ± 1.3 mg/dL in the older men (*P* = 0.336 between groups). At the end of the study, serum ascorbic acid was 11.4 ± 0.5 mg/dL in the young men and 11.4 ± 0.7 mg/dL in the older men (*P* = 0.982 between groups). Expectedly, intravenous infusion of ascorbic acid resulted in a 10 to 20‐fold increase in serum levels of ascorbic acid.

Despite the high antioxidant load, the eight older men who received both saline and ascorbic acid had very similar physiological responses to 5 min of breathing 10% oxygen under both infusions. As expected, HR and MV increased in response to 5 min of breathing 10% oxygen under both infusions, whereas SaO2 decreased and EtCO2 also decreased (Table 2). However, as shown in Figure 2 (right panel), there was no treatment by time interactions for any of the cardiovascular variables. Thus, intravenous ascorbic acid did not alter the physiological response to continuous hypoxia in older men. There was also no effect of ascorbic acid in the young men (Table 2). Consistent with Experiment 1, there was a blunted tachycardia and blunted forearm vasodilation when comparing the five younger men to the eight older men under both treatments.

**Table 2.** Physiological responses to continuous hypoxia (10% oxygen, 90% nitrogen) in Experiment 2.

	Base	1 min	2 min	3 min	4 min	5 min	Infuse	Time	Age
HR (bpm)									
Young NSS	55 ± 3	62 ± 6	66 ± 5	71 ± 5	75 ± 3	80 ± 5	0.001	<0.001	0.048
Older NSS	55 ± 3	58 ± 3	59 ± 3	61 ± 3	63 ± 3	64 ± 3			
Young Vit C	58 ± 3	67 ± 3	74 ± 2	80 ± 4	80 ± 3	82 ± 5			
Older Vit C	57 ± 3	60 ± 3	63 ± 3	64 ± 3	68 ± 4	68 ± 3			
MAP (mmHg)									
Young NSS	79 ± 7	81 ± 8	80 ± 8	76 ± 9	77 ± 8	78 ± 7	0.877	0.402	0.045
Older NSS	92 ± 4	94 ± 4	91 ± 4	91 ± 4	91 ± 5	92 ± 5			
Young Vit C	78 ± 6	77 ± 7	78 ± 6	78 ± 6	77 ± 6	75 ± 6			
Older Vit C	92 ± 2	95 ± 3	96 ± 4	93 ± 4	94 ± 4	94 ± 4			
FBF (mL/min)									
Young NSS	33 ± 5	39 ± 6	44 ± 8	44 ± 8	44 ± 8	41 ± 7	0.956	0.014	0.008
Older NSS	22 ± 4	21 ± 4	18 ± 3	21 ± 4	21 ± 3	25 ± 4			
Young Vit C	33 ± 4	35 ± 5	38 ± 5	38 ± 6	40 ± 4	44 ± 7			
Older Vit C	24 ± 3	23 ± 2	24 ± 3	23 ± 4	25 ± 4	25 ± 3			
CVC (au)									
Young NSS	100 ± 0	107 ± 5	118 ± 3	150 ± 10	147 ± 6	143 ± 6	0.253	<0.001	0.068
Older NSS	100 ± 0	96 ± 4	104 ± 6	117 ± 9	117 ± 9	122 ± 6			
Young Vit C	100 ± 0	97 ± 5	114 ± 10	125 ± 12	136 ± 11	147 ± 10			
Older Vit C	100 ± 0	95 ± 3	99 ± 7	116 ± 6	118 ± 9	120 ± 7			
SaO2 (%)									
Young NSS	97 ± 0.5	92 ± 0.9	88 ± 0.6	84 ± 0.9	83 ± 1.8	82 ± 2.4	0.291	<0.001	0.218
Older NSS	97 ± 0.3	93 ± 0.9	90 ± 1.0	88 ± 1.3	84 ± 1.5	83 ± 1.6			
Young Vit C	98 ± 0.3	82 ± 1.1	88 ± 0.3	84 ± 0.8	82 ± 1.5	80 ± 1.5			
Older Vit C	97 ± 0.3	93 ± 0.6	88 ± 0.8	86 ± 0.8	84 ± 0.9	81 ± 1.3			
EtCO2 (mmHg)									
Young NSS	42 ± 1.6	42 ± 1.2	40 ± 1.5	41 ± 1.8	40 ± 1.8	40 ± 1.5	0.362	<0.001	0.003
Older NSS	35 ± 1.4	34 ± 1.5	34 ± 1.4	34 ± 1.5	33 ± 1.1	33 ± 1.1			
Young Vit C	41 ± 1.8	41 ± 1.1	41 ± 1.1	41 ± 1.0	40 ± 0.6	39 ± 0.9			
Older Vit C	34 ± 1.2	33 ± 1.3	33 ± 1.1	32 ± 1.1	32 ± 1.1	32 ± 1.2			
MV (L/min)									
Young NSS	8.7 ± 0.9	11.3 ± 1.2	11.9 ± 1.2	10.2 ± 0.8	11.8 ± 1.7	11.4 ± 1.6	0.626	<0.001	0.998
Older NSS	9.9 ± 0.8	10.8 ± 0.9	10.3 ± 1.0	10.2 ± 0.7	12.1 ± 0.6	11.5 ± 0.6			
Young Vit C	9.9 ± 1.1	11.3 ± 0.7	11.3 ± 1.6	11.1 ± 1.8	11.7 ± 0.7	11.4 ± 0.9			
Older Vit C	9.3 ± 0.7	11.3 ± 0.8	11.0 ± 0.8	11.5 ± 1.0	11.0 ± 0.6	11.7 ± 0.5			

In Experiment 2, eight older men and five young men were exposed to 5 min of continuously breathing 10% oxygen (hypoxia) while being infused both normal saline solution (NSS) and ascorbic acid (Vit C). Ascorbic acid had no significant effect on any of the measured variables.

In response to 5 min of breathing 21% oxygen (i.e., room air), there were no main effects for infusion or time (Table 3). However, the older men had higher MAP, lower FBF and lower EtCO2 compared to young men at all time points.

**Table 3.** Physiologic responses to normoxia (21% oxygen) in Experiment 2.

	Base	1 min	2 min	3 min	4 min	5 min	Infuse	Time	Age
HR (bpm)									
Young NSS	57 ± 3	57 ± 4	56 ± 4	56 ± 4	56 ± 5	56 ± 5	0.547	0.334	0.920
Older NSS	55 ± 3	54 ± 2	56 ± 3	54 ± 3	55 ± 3	59 ± 3			
Young Vit C	54 ± 4	55 ± 4	56 ± 3	55 ± 3	53 ± 3	54 ± 3			
Older Vit C	56 ± 3	55 ± 2	54 ± 2	55 ± 2	57 ± 2	57 ± 2			
MAP (mmHg)									
Young NSS	79 ± 6	80 ± 6	80 ± 6	81 ± 6	80 ± 7	80 ± 7	0.239	0.129	0.038
Older NSS	91 ± 2	82 ± 3	91 ± 3	94 ± 3	94 ± 3	91 ± 3			
Young Vit C	79 ± 6	75 ± 7	77 ± 6	82 ± 7	77 ± 6	78 ± 7			
Older Vit C	88 ± 4	88 ± 4	88 ± 4	89 ± 4	91 ± 4	94 ± 1			
FBF (mL/min)									
Young NSS	39 ± 5	40 ± 7	38 ± 6	39 ± 7	37 ± 6	36 ± 2	0.35	0.136	0.002
Older NSS	20 ± 3	16 ± 2	16 ± 2	15 ± 2	16 ± 2	20 ± 3			
Young Vit C	36 ± 4	41 ± 4	41 ± 3	38 ± 3	42 ± 3	39 ± 5			
Older Vit C	25 ± 4	21 ± 4	24 ± 4	21 ± 4	23 ± 4	23 ± 4			
CVC (au)									
Young NSS	100 ± 0	109 ± 8	100 ± 3	104 ± 3	107 ± 7	109 ± 7	0.848	0.121	0.364
Older NSS	100 ± 0	105 ± 4	96 ± 4	94 ± 7	99 ± 6	105 ± 6			
Young Vit C	100 ± 0	109 ± 5	103 ± 8	91 ± 4	111 ± 7	110 ± 8			
Older Vit C	100 ± 0	100 ± 5	99 ± 3	98 ± 2	101 ± 6	96 ± 6			
SaO2 (%)									
Young NSS	98 ± 0.1	98 ± 0.1	98 ± 0.3	98 ± 0.3	98 ± 0.1	98 ± 0.3	0.368	0.111	0.07
Older NSS	97 ± 0.4	98 ± 0.4	97 ± 0.4	97 ± 0.3	97 ± 0.3	98 ± 0.3			
Young Vit C	98 ± 0.3	98 ± 0.3	98 ± 0.5	97 ± 0.5	98 ± 0.5	98 ± 0.3			
Older Vit C	97 ± 0.2	97 ± 0.3	97 ± 0.2	97 ± 0.1	97 ± 0.2	97 ± 0.2			
EtCO2 (mmHg)									
Young NSS	43 ± 0.6	43 ± 1.1	43 ± 0.3	43 ± 0.4	43 ± 0.5	44 ± 0.5	0.52	0.007	0.002
Older NSS	36 ± 1.4	36 ± 1.4	36 ± 1.4	36 ± 1.5	37 ± 1.6	37 ± 1.5			
Young Vit C	42 ± 0.8	41 ± 0.4	41 ± 0.3	44 ± 0.3	43 ± 0.8	42 ± 0.8			
Older Vit C	35 ± 0.8	35 ± 1.0	36 ± 1.0	36 ± 1.0	36 ± 1.1	37 ± 1.3			
MV (L/min)									
Young NSS	8.8 ± 0.8	9.2 ± 0.4	9.6 ± 0.5	9.1 ± 0.4	9.3 ± 0.8	8.7 ± 0.9	0.881	0.683	0.689
Older NSS	9.0 ± 0.6	8.7 ± 0.6	9.2 ± 0.6	8.5 ± 0.7	9.4 ± 1.0	8.9 ± 0.5			
Young Vit C	10.1 ± 0.7	10.2 ± 1.0	10.7 ± 0.6	9.8 ± 0.9	9.5 ± 0.7	9.3 ± 0.7			
Older Vit C	10.2 ± 0.9	9.2 ± 0.8	10.2 ± 0.9	9.3 ± 1.0	10.2 ± 0.7	9.1 ± 0.6			

In Experiment 2, eight older men and five young men were exposed to 5 min of continuously breathing 21% oxygen (normoxia or room air) while being infused both normal saline solution (NSS) and ascorbic acid (Vit C). Ascorbic acid had no significant effect on any of the measured variables.

## Discussion

The purpose of this study was to determine the effect of aging on FVC and CVC responses to hypoxia and whether acute systemic infusion of ascorbic acid could modify these responses in older men. Consistent with our hypothesis, older men had an attenuated FVC response compared to young men when exposed to 5 min of hypoxia but CVC responses were comparable between groups. To our knowledge, this is the first evidence that hypoxia‐induced vasodilation is different between skin and muscle. The data from Experiment 2 do not support our original hypothesis that ascorbic acid would enhance hypoxia‐induced vasodilation in older men. These findings advance our basic understanding of how aging influences vascular responses to reduced blood oxygen levels and suggest that in healthy humans hypoxia‐induced vasodilation is not restrained by reactive oxygen species.

Exposure to hypoxia leads to a reduction in SaO2 and activation of the arterial (peripheral) chemoreflex which then stimulates ventilation and raises sympathetic outflow to skeletal muscle and the heart (Halliwill 2003). The current data are consistent with previous studies that demonstrated a blunted tachycardia in the older subjects in response to short‐duration hypoxia (Kronenberg and Drage 1973; Davy et al. 1997; Lhuissier et al. 2012). As expected, MAP was higher in the older men at all time points but was unaffected by 5 min of hypoxia. Respiratory responses were also not significantly influenced by age or ascorbic acid. Importantly, we demonstrated that FBF and FVC responses to hypoxia were attenuated in the older men. These findings are consistent with prior studies by Kirby et al. (2012) and Kravec et al. (1972). However, the current data are contrary to recent work by Casey et al. (2011) and Limberg et al. (2012); these two studies enrolled both men and women. Some potential reasons for the impaired FBF and FVC in older men include (1) enhanced sympathetic vasoconstrictor responses to hypoxia in the older men, (2) attenuated beta‐2 adrenergic vasodilation in the older men, (3) attenuated nitric oxide production in the older men (either due to aging per se or due to the attenuated HR response and resultant reduced shear stress release of nitric oxide), (4) alterations in other metabolites that respond to hypoxia in older men, and (5) augmented oxidative stress in the older men. These five reasons are discussed below.

First, aging is associated with increased SNS activity (Sundlof and Wallin 1978; Narkiewicz et al. 2005). However, changes in MSNA in response to acute hypoxia do not appear to be enhanced in older men (Davy et al. 1997). Richards et al. (2013) recently demonstrated elevated alpha‐adrenergic vasoconstriction in response to hypoxia in older men but they suggested that several factors are likely responsible for an impaired vasodilatory response with aging. Second, Richards et al. (2013) found that beta‐adrenergic vasodilation was attenuated in the aging population in response to hypoxia. Several studies have shown that up to 50% of hypoxia‐induced vasodilation is mediated by beta‐adrenergic receptors (Blauw et al. 1995; Weisbrod et al. 2001; Wilkins et al. 2008) and beta‐adrenergic receptors clearly become less responsive to agonists with age (Xiao and Lakatta 1992). Third, it has been demonstrated that nitric oxide (NO) signaling is a major contributor to the compensatory vasodilation at rest (Casey et al. 2010) and also that it is blunted in older individuals under hypoxic conditions (Casey et al. 2011). Indeed, blockade of NO production with L‐NMMA decreases the hypoxic forearm vasodilatory response in young adults (Blitzer et al. 1996; Casey et al. 2010). This indicates that hypoxic vasodilation requires functionally intact endothelium and subsequent NO release. Casey et al. (2011) showed blunted NO signaling in older adults when compared to younger adults during hypoxic exercise while others have shown blunted NO responses to acetylcholine (Taddei et al. 2001) and flow‐ mediated dilation (Heiss et al. 2005) between these groups. Fourth, several other local vasodilatory signaling factors could play a significant role in hypoxia‐induced vasodilation including adenosine (Carlsson et al. 1987; Leuenberger et al. 1999), prostaglandins (Carlsson et al. 1987; Engelke et al. 1996), release of ATP from red blood cells (Kirby et al. 2012) and local myogenic factors (Carlsson et al. 1987; Schubert and Mulvany 1999; Koller and Bagi 2002); however, limited data exists on the role of these mechanisms on hypoxia‐induced vasodilation with aging.

Fifth, it is clear that aging is associated with heightened oxidative stress and impaired antioxidant defense (Sohal and Weindruch 1996). Physiological studies have demonstrated that ascorbic acid can acutely modify vascular responses in groups of people with elevated oxidative stress. Indeed, Taddei et al. (2001) showed that intra‐arterial ascorbic acid infusion increased vasodilation in response to acetylcholine in older subjects and Holowatz et al. (2006) demonstrated that acute intradermal ascorbate increased cutaneous vasodilation in the aged skin in response to whole body heating. We recently demonstrated that ascorbic acid (identical dose as in the current study) blocks the forearm vasoconstrictor response to 100% oxygen (i.e., an acute pro‐oxidant) and also normalizes exercise blood pressure in patients with peripheral arterial disease (i.e., a chronic condition characterized by high levels of systemic oxidative stress) (Muller et al. 2012, 2013a). Additionally, Eskurza et al. (2004) demonstrated that intravenous ascorbic acid enhanced flow‐mediated forearm vasodilation in older men. Based on these previous studies and the fact that part of age‐related impairments in hypoxia‐induced vasodilation is endothelium dependent (Blitzer et al. 1996; Casey et al. 2010), we reasoned that ascorbic acid would enhance hypoxia‐induced vasodilation in the older men. Nevertheless, the current data (Experiment 2) suggest that oxidative stress does not play a significant role in hypoxia‐induced vasodilation because high‐ dose ascorbic (raising serum levels 10–20 fold above baseline) acid had no effect on vasodilator responses in young or older men. Our findings are consistent with a recent study that found no effect of intra‐arterial ascorbic acid on hypoxia‐induced vasodilation in heart failure (Nazare Nunes Alves et al. 2012). Taken together, the regulation of skeletal muscle blood flow during hypoxia involves these five factors listed above and there is considerable redundancy such that the alteration of one pathway due to aging may influence other pathways.

To our knowledge, no prior study has evaluated how hypoxia influences CVC in older adults. Our data, while not significant between groups, contribute importantly to the literature because previous work has found age impairments in the regulation of skin blood flow in response to heat (Kenney et al. 1997; Minson et al. 2002) and cold stress (Kenney and Armstrong 1996). Moreover, our CVC data suggest that age impairments in hypoxia‐induced vasodilation are present with regard to muscle blood flow but not skin blood flow. We speculate that cutaneous vasodilation in response to hypoxia is not simply a secondary effect of increased whole limb blood flow.

### Experimental considerations

There are five main issues we would like to point out that may affect the interpretation of these data. First, we conducted acute studies and since aging is a chronic process, it is possible that the impairments in vascular function cannot be acutely altered (e.g., products of oxidative stress may chronically alter carotid body chemosensitive afferents or efferent nerve activity). Second, the forearm receives a relatively small percentage of cardiac output and we may have been able to detect larger effects had we measured the femoral artery instead of the brachial arterial or used a longer duration of hypoxia. Third, although blood sampling did detect commonly observed age‐related differences in LDL cholesterol (Miller 1984), hs‐CRP (Woloshin and Schwartz 2005), and fibrinogen (Laharrague et al. 1993; Hager et al. 1994; Fu and Nair 1998) the older men in our study were quite healthy. Therefore, it is possible that people with more cardiovascular risk factors would show different responses than these older, healthy men (i.e. oxidative stress may play a bigger role in older, less healthy men). To support this theory, the young men in this experiment, with presumably healthy endothelial function showed no change to ascorbic acid as well. Fourth, maximal skin blood flow is reduced in older adults (Holowatz et al. 2010) and since we did not perform local heating, the CVC data presented in this study are not normalized to each individual's maximum skin blood flow (i.e., the older adults were operating at a higher relative percent of maximum). Fifth, it is possible that ascorbic acid did not affect the ROS species that are important in the age‐impairment in skeletal muscle hypoxia‐induced vasodilation.

### Clinical implications

Attenuated vasodilator responses in the forearm may translate to attenuated responses in other vascular beds. For instance, there may be an increased risk of myocardial injury upon exposure to acute hypoxia in older adults. Furthermore, patients with chronic obstructive pulmonary disease and obstructive sleep apnea (i.e., chronic intermittent hypoxia) tend to be older so it is likely that the cardiovascular complications of these diseases are also influenced by the aging process. However, our findings question whether age‐related impairments in muscle (or coronary) blood flow can be modified by acute administration of ascorbic acid. Moreover, these data highlight that skin and muscle blood flow responses to hypoxia are differentially affected by the aging process.

## Conclusions

Healthy older men have a blunted hypoxia‐induced vasodilation in forearm skeletal muscle compared to young men but forearm skin blood flow responses were comparable between groups. The age‐related impairment in hypoxia‐induced vasodilation was not altered by systemic infusion of ascorbic acid which suggests that oxidative stress does not play a mechanistic role in this process. Future studies may enroll older patients with cardiopulmonary disease to further investigate hypoxia‐induced vasodilation in different vascular beds.

## Acknowledgments

The authors are grateful for the nursing support provided by Cheryl Blaha, Jessica Mast, Todd Nicklas, and Aimee Caufmann and the engineering support provided by Dr. Michael Herr. Our gratitude is also extended to Anne Muller for preparing the graphics for this study and to Dr. Lawrence Sinoway for clinical oversight and funding support. Finally, the authors acknowledge the administrative guidance of Kris Gray and Jen Stoner.

## Conflict of Interest

None declared.

## References

[b1] Blauw G. J. Westendorp R. G. Simons M. Chang P. C. Frolich M. Meinders A. E. 1995 beta‐Adrenergic receptors contribute to hypoxaemia induced vasodilation in man. Br. J. Clin. Pharmacol.; 40:453-458. 8703649 PMC1365191

[b2] Blitzer M. L. Lee S. D. Creager M. A. 1996 Endothelium‐derived nitric oxide mediates hypoxic vasodilation of resistance vessels in humans. Am. J. Physiol.; 271:H1182-H1185. 8853358 10.1152/ajpheart.1996.271.3.H1182

[b3] Carlsson I. Sollevi A. Wennmalm A. 1987 The role of myogenic relaxation, adenosine and prostaglandins in human forearm reactive hyperaemia. J. Physiol.; 389:147-161. 3681724 10.1113/jphysiol.1987.sp016651 PMC1192075

[b4] Casey D. P. Madery B. D. Curry T. B. Eisenach J. H. Wilkins B. W. Joyner M. J. 2010 Nitric oxide contributes to the augmented vasodilatation during hypoxic exercise. J. Physiol.; 588:373-385. 19948661 10.1113/jphysiol.2009.180489 PMC2821731

[b5] Casey D. P. Walker B. G. Curry T. B. Joyner M. J. 2011 Ageing reduces the compensatory vasodilatation during hypoxic exercise: the role of nitric oxide. J. Physiol.; 589:1477-1488. 21282292 10.1113/jphysiol.2010.203539 PMC3082105

[b6] Casey D. P. Shepherd J. R. Joyner M. J. 2013 Sex and vasodilator responses to hypoxia at rest and during exercise. J. Appl. Physiol. (1985); 116:927-936. 23823148 10.1152/japplphysiol.00409.2013 PMC3972743

[b7] Celermajer D. S. Sorensen K. E. Spiegelhalter D. J. Georgakopoulos D. Robinson J. Deanfield J. E. 1994 Aging is associated with endothelial dysfunction in healthy men years before the age‐related decline in women. J. Am. Coll. Cardiol.; 24:471-476. 8034885 10.1016/0735-1097(94)90305-0

[b8] Davy K. P. Jones P. P. Seals D. R. 1997 Influence of age on the sympathetic neural adjustments to alterations in systemic oxygen levels in humans. Am. J. Physiol.; 273:R690-R695. 9277556 10.1152/ajpregu.1997.273.2.R690

[b9] DeLorey D. S. Shaw C. N. Shoemaker J. K. Kowalchuk J. M. Paterson D. H. 2004 The effect of hypoxia on pulmonary O_2_ uptake, leg blood flow and muscle deoxygenation during single‐leg knee‐extension exercise. Exp. Physiol.; 89:293-302. 15123565 10.1113/expphysiol.2003.026864

[b10] Engelke K. A. Halliwill J. R. Proctor D. N. Dietz N. M. Joyner M. J. 1996 Contribution of nitric oxide and prostaglandins to reactive hyperemia in human forearm. J. Appl. Physiol. (1985); 81:1807-1814. 8904603 10.1152/jappl.1996.81.4.1807

[b11] Eskurza I. Monahan K. D. Robinson J. A. Seals D. R. 2004 Effect of acute and chronic ascorbic acid on flow‐mediated dilatation with sedentary and physically active human ageing. J. Physiol.; 556:315-324. 14754992 10.1113/jphysiol.2003.057042 PMC1664895

[b12] Frei B. 1991 Ascorbic acid protects lipids in human plasma and low‐density lipoprotein against oxidative damage. Am. J. Clin. Nutr.; 54:1113S-1118S. 1962556 10.1093/ajcn/54.6.1113s

[b13] Fu A. Nair K. S. 1998 Age effect on fibrinogen and albumin synthesis in humans. Am. J. Physiol.; 275:E1023-E1030. 9843745 10.1152/ajpendo.1998.275.6.E1023

[b14] Gryglewski R. J. Palmer R. M. Moncada S. 1986 Superoxide anion is involved in the breakdown of endothelium‐derived vascular relaxing factor. Nature; 320:454-456. 3007998 10.1038/320454a0

[b15] Hager K. Felicetti M. Seefried G. Platt D. 1994 Fibrinogen and aging. Aging; 6:133-138. 7918730 10.1007/BF03324226

[b16] Halliwill J. R. 2003 Hypoxic regulation of blood flow in humans. Skeletal muscle circulation and the role of epinephrine. Adv. Exp. Med. Biol.; 543:223-236. 14713125

[b17] Heiss C. Keymel S. Niesler U. Ziemann J. Kelm M. Kalka C. 2005 Impaired progenitor cell activity in age‐related endothelial dysfunction. J. Am. Coll. Cardiol.; 45:1441-1448. 15862416 10.1016/j.jacc.2004.12.074

[b18] Heistad D. D. Wheeler R. C. 1970 Effect of acute hypoxia on vascular responsiveness in man. I. Responsiveness to lower body negative pressure and ice on the forehead. II. Responses to norepinephrine and angiotensin. 3. Effect of hypoxia and hypocapnia. J. Clin. Invest.; 49:1252-1265. 4316225 10.1172/JCI106338 PMC322590

[b19] Herr M. D. Hogeman C. S. Koch D. W. Krishnan A. Momen A. Leuenberger U. A. 2010 A real‐time device for converting Doppler ultrasound audio signals into fluid flow velocity. Am. J. Physiol. Heart Circ. Physiol.; 298:H1626-H1632. 20173048 10.1152/ajpheart.00713.2009 PMC2867441

[b20] Holowatz L. A. Thompson C. S. Kenney W. L. 2006 Acute ascorbate supplementation alone or combined with arginase inhibition augments reflex cutaneous vasodilation in aged human skin. Am. J. Physiol. Heart Circ. Physiol.; 291:H2965-H2970. 16905599 10.1152/ajpheart.00648.2006

[b21] Holowatz L. A. Thompson‐Torgerson C. S. Kenney W. L. 2007 Altered mechanisms of vasodilation in aged human skin. Exerc. Sport Sci. Rev.; 35:119-125. 17620930 10.1097/jes.0b013e3180a02f85

[b22] Holowatz L. A. Thompson‐Torgerson C. Kenney W. L. 2010 Aging and the control of human skin blood flow. Front. Biosci.; 15:718-739. 10.2741/3642 PMC3228253 20036842

[b23] Katusic Z. S. Vanhoutte P. M. 1989 Superoxide anion is an endothelium‐derived contracting factor. Am. J. Physiol.; 257:H33-H37. 2546450 10.1152/ajpheart.1989.257.1.H33

[b24] Kenney W. L. Armstrong C. G. 1996 Reflex peripheral vasoconstriction is diminished in older men. J. Appl. Physiol.; 80:512-515. 8929592 10.1152/jappl.1996.80.2.512

[b25] Kenney W. L. Morgan A. L. Farquhar W. B. Brooks E. M. Pierzga J. M. Derr J. A. 1997 Decreased active vasodilator sensitivity in aged skin. Am. J. Physiol.; 272:H1609-H1614. 9139942 10.1152/ajpheart.1997.272.4.H1609

[b26] Kirby B. S. Voyles W. F. Simpson C. B. Carlson R. E. Schrage W. G. Dinenno F. A. 2009 Endothelium‐dependent vasodilatation and exercise hyperaemia in ageing humans: impact of acute ascorbic acid administration. J. Physiol.; 587:1989-2003. 19307300 10.1113/jphysiol.2008.167320 PMC2689338

[b27] Kirby B. S. Crecelius A. R. Voyles W. F. Dinenno F. A. 2012 Impaired skeletal muscle blood flow control with advancing age in humans: attenuated ATP release and local vasodilation during erythrocyte deoxygenation. Circ. Res.; 111:220-230. 22647875 10.1161/CIRCRESAHA.112.269571 PMC3393524

[b28] Koller A. Bagi Z. 2002 On the role of mechanosensitive mechanisms eliciting reactive hyperemia. Am. J. Physiol. Heart Circ. Physiol.; 283:H2250-H2259. 12427591 10.1152/ajpheart.00545.2002

[b29] Kravec T. F. Eggers G. W. Jr Kettel L. J. 1972 Influence of patient age on forearm and systemic vascular response to hypoxaemia. Clin. Sci.; 42:555-565. 5032887 10.1042/cs0420555

[b30] Kronenberg R. S. Drage C. W. 1973 Attenuation of the ventilatory and heart rate responses to hypoxia and hypercapnia with aging in normal men. J. Clin. Invest.; 52:1812-1819. 4719663 10.1172/JCI107363 PMC302461

[b31] Laharrague P. F. Cambus J. P. Fillola G. Corberand J. X. 1993 Plasma fibrinogen and physiological aging. Aging; 5:445-449. 8161576 10.1007/BF03324200

[b32] Leuenberger U. Gleeson K. Wroblewski K. Prophet S. Zelis R. Zwillich C. 1991 Norepinephrine clearance is increased during acute hypoxemia in humans. Am. J. Physiol.; 261:H1659-H1664. 1951753 10.1152/ajpheart.1991.261.5.H1659

[b33] Leuenberger U. A. Gray K. Herr M. D. 1999 Adenosine contributes to hypoxia‐induced forearm vasodilation in humans. J. Appl. Physiol. (1985); 87:2218-2224. 10601170 10.1152/jappl.1999.87.6.2218

[b34] Leuenberger U. A. Hardy J. C. Herr M. D. Gray K. S. Sinoway L. I. 2001 Hypoxia augments apnea‐induced peripheral vasoconstriction in humans. J. Appl. Physiol.; 90:1516-1522. 11247954 10.1152/jappl.2001.90.4.1516

[b35] Lhuissier F. J. Canoui‐Poitrine F. Richalet J. P. 2012 Ageing and cardiorespiratory response to hypoxia. J. Physiol.; 590:5461-5474. 22907053 10.1113/jphysiol.2012.238527 PMC3515831

[b36] Limberg J. K. Evans T. D. Pegelow D. F. Eldridge M. W. Sebranek J. J. Proctor L. T. 2012 Heterogeneous vascular responses to hypoxic forearm exercise in young and older adults. Eur. J. Appl. Physiol.; 112:3087-3095. 22198326 10.1007/s00421-011-2280-x PMC4158744

[b37] Miller N. E. 1984 Why does plasma low density lipoprotein concentration in adults increase with age? Lancet; 1:263-267. 6143004 10.1016/s0140-6736(84)90135-1

[b38] Minson C. T. Holowatz L. A. Wong B. J. Kenney W. L. Wilkins B. W. 2002 Decreased nitric oxide‐ and axon reflex‐mediated cutaneous vasodilation with age during local heating. J. Appl. Physiol. (1985); 93:1644-1649. 12381749 10.1152/japplphysiol.00229.2002

[b39] Monahan K. D. Eskurza I. Seals D. R. 2004 Ascorbic acid increases cardiovagal baroreflex sensitivity in healthy older men. Am. J. Physiol. Heart Circ. Physiol.; 286:H2113-H2117. 14962830 10.1152/ajpheart.01054.2003

[b40] Moradkhan R. McQuillan P. Hogeman C. Leuenberger A. Linton‐Frazier L. Leuenberger U. A. 2007 Metabolic forearm vasodilation is enhanced following Bier block with phentolamine. Am. J. Physiol. Heart Circ. Physiol.; 293:H2289-H2295. 17675565 10.1152/ajpheart.01422.2006

[b41] Moradkhan R. Spitnale B. McQuillan P. Hogeman C. Gray K. S. Leuenberger U. A. 2010 Hypoxia‐induced vasodilation and effects of regional phentolamine in awake patients with sleep apnea. J. Appl. Physiol. (1985); 108:1234-1240. 20223993 10.1152/japplphysiol.90855.2008 PMC2867527

[b42] Muller M. D. Drew R. C. Blaha C. A. Mast J. L. Cui J. Reed A. B. 2012 Oxidative stress contributes to the augmented exercise pressor reflex in peripheral arterial disease patients. J. Physiol.; 590:6237-6246. 23006479 10.1113/jphysiol.2012.241281 PMC3530129

[b43] Muller M. D. Drew R. C. Cui J. Blaha C. A. Mast J. L. Sinoway L. I. 2013a Effect of oxidative stress on sympathetic and renal vascular responses to ischemic exercise. Physiol. Rep.; 1:1-13. 10.1002/phy2.47 PMC3787721 24098855

[b44] Muller M. D. Mast J. L. Patel H. Sinoway L. I. 2013b Cardiac mechanics are impaired during fatiguing exercise and cold pressor test in healthy older adults. J. Appl. Physiol. (1985); 114:186-194. 23154996 10.1152/japplphysiol.01165.2012 PMC3544501

[b45] Muller M. D. Sauder C. L. Ray C. A. 2013c Melatonin attenuates the skin sympathetic nerve response to mental stress. Am. J. Physiol. Heart Circ. Physiol.; 305:H1382-H1386. 23997106 10.1152/ajpheart.00470.2013 PMC3840247

[b46] Muller M. D. Sauder C. L. Ray C. A. 2013d Mental stress elicits sustained and reproducible increases in skin sympathetic nerve activity. Physiol. Rep.; 1:1-8. 10.1002/phy2.2 PMC3673729 23750321

[b47] Narkiewicz K. Phillips B. G. Kato M. Hering D. Bieniaszewski L. Somers V. K. 2005 Gender‐selective interaction between aging, blood pressure, and sympathetic nerve activity. Hypertension; 45:522-525. 15767469 10.1161/01.HYP.0000160318.46725.46

[b48] Nazare Nunes Alves M. J. dos Santos M. R. Nobre T. S. Martinez D. G. Pereira Barretto A. C. Brum P. C. 2012 Mechanisms of blunted muscle vasodilation during peripheral chemoreceptor stimulation in heart failure patients. Hypertension; 60:669-676. 22802226 10.1161/HYPERTENSIONAHA.112.195776

[b49] Newcomer S. C. Leuenberger U. A. Hogeman C. S. Proctor D. N. 2005 Heterogeneous vasodilator responses of human limbs: influence of age and habitual endurance training. Am. J. Physiol. Heart Circ. Physiol.; 289:H308-H315. 15778285 10.1152/ajpheart.01151.2004

[b50] Patel H. M. Heffernan M. J. Ross A. J. Muller M. D. 2014 Sex differences in forearm vasoconstrictor response to voluntary apnea. Am. J. Physiol. Heart Circ. Physiol.; 306:H309-H316. 24322616 10.1152/ajpheart.00746.2013 PMC3920136

[b51] Richards J. C. Crecelius A. R. Kirby B. S. Garcia L. J. Luckasen G. J. Larson D. G. 2013 Impaired hypoxic vasodilation in healthy older adults: role for altered sympatho‐adrenal control of vascular tone. FASEB J.; 27:1119.1

[b52] Richardson D. W. Kontos H. A. Raper A. J. Patterson J. L. Jr 1967 Modification by beta‐adrenergic blockade of the circulatory respones to acute hypoxia in man. J. Clin. Invest.; 46:77-85. 4381183 10.1172/JCI105513 PMC297022

[b53] Rowell L. B. Johnson D. G. Chase P. B. Comess K. A. Seals D. R. 1989 Hypoxemia raises muscle sympathetic activity but not norepinephrine in resting humans. J. Appl. Physiol. (1985); 66:1736-1743. 2732164 10.1152/jappl.1989.66.4.1736

[b54] Saito M. Mano T. Iwase S. Koga K. Abe H. Yamazaki Y. 1988 Responses in muscle sympathetic activity to acute hypoxia in humans. J. Appl. Physiol. (1985); 65:1548-1552. 3182518 10.1152/jappl.1988.65.4.1548

[b55] Schubert R. Mulvany M. J. 1999 The myogenic response: established facts and attractive hypotheses. Clin. Sci. (Lond.); 96:313-326. 10087237

[b56] Simmons G. H. Minson C. T. Cracowski J. L. Halliwill J. R. 2007 Systemic hypoxia causes cutaneous vasodilation in healthy humans. J. Appl. Physiol. (1985); 103:608-615. 17510298 10.1152/japplphysiol.01443.2006

[b57] Sohal R. S. Weindruch R. 1996 Oxidative stress, caloric restriction, and aging. Science; 273:59-63. 8658196 10.1126/science.273.5271.59 PMC2987625

[b58] Somers V. K. Mark A. L. Zavala D. C. Abboud F. M. 1989 Contrasting effects of hypoxia and hypercapnia on ventilation and sympathetic activity in humans. J. Appl. Physiol. (1985); 67:2101-2106. 2513316 10.1152/jappl.1989.67.5.2101

[b59] Sundlof G. Wallin B. G. 1978 Human muscle nerve sympathetic activity at rest. Relationship to blood pressure and age. J. Physiol.; 274:621-637. 625012 10.1113/jphysiol.1978.sp012170 PMC1282513

[b60] Taddei S. Virdis A. Ghiadoni L. Magagna A. Salvetti A. 1998 Vitamin C improves endothelium‐dependent vasodilation by restoring nitric oxide activity in essential hypertension. Circulation; 97:2222-2229. 9631871 10.1161/01.cir.97.22.2222

[b61] Taddei S. Virdis A. Ghiadoni L. Salvetti G. Bernini G. Magagna A. 2001 Age‐related reduction of NO availability and oxidative stress in humans. Hypertension; 38:274-279. 11509489 10.1161/01.hyp.38.2.274

[b62] Tew G. A. George K. P. Cable N. T. Hodges G. J. 2012 Endurance exercise training enhances cutaneous microvascular reactivity in post‐menopausal women. Microvasc. Res.; 83:223-228. 21945654 10.1016/j.mvr.2011.09.002

[b63] Weisbrod C. J. Minson C. T. Joyner M. J. Halliwill J. R. 2001 Effects of regional phentolamine on hypoxic vasodilatation in healthy humans. J. Physiol.; 537:613-621. 11731591 10.1111/j.1469-7793.2001.00613.x PMC2278971

[b64] Wilkins B. W. Pike T. L. Martin E. A. Curry T. B. Ceridon M. L. Joyner M. J. 2008 Exercise intensity‐dependent contribution of beta‐adrenergic receptor‐mediated vasodilatation in hypoxic humans. J. Physiol.; 586:1195-1205. 18048452 10.1113/jphysiol.2007.144113 PMC2375634

[b65] Wilson T. E. Sauder C. L. Kearney M. L. Kuipers N. T. Leuenberger U. A. Monahan K. D. 2007 Skin‐surface cooling elicits peripheral and visceral vasoconstriction in humans. J. Appl. Physiol.; 103:1257-1262. 17673561 10.1152/japplphysiol.00401.2007

[b66] Woloshin S. Schwartz L. M. 2005 Distribution of C‐reactive protein values in the United States. N. Engl. J. Med.; 352:1611-1613. 15829550 10.1056/NEJM200504143521525

[b67] Xiao R. P. Lakatta E. G. 1992 Deterioration of beta‐adrenergic modulation of cardiovascular function with aging. Ann. N. Y. Acad. Sci.; 673:293-310. 1336647 10.1111/j.1749-6632.1992.tb27465.x

[b68] Xie A. Skatrud J. B. Puleo D. S. Morgan B. J. 2001 Exposure to hypoxia produces long‐lasting sympathetic activation in humans. J. Appl. Physiol. (1985); 91:1555-1562. 11568136 10.1152/jappl.2001.91.4.1555

